# Prevalence of scoliosis in children and adolescents: a systematic review and meta-analysis

**DOI:** 10.3389/fped.2024.1399049

**Published:** 2024-07-23

**Authors:** Mingyang Li, Qilong Nie, Jiaying Liu, Zeping Jiang

**Affiliations:** ^1^ The Eighth Clinical Medical College, Guangzhou University of Chinese Medicine, Foshan, Guangdong, China; ^2^ Foshan Hospital of Traditional Chinese Medicine, Guangzhou University of Chinese Medicine, Foshan, Guangdong, China

**Keywords:** scoliosis, children, adolescents, prevalence, risk factors, systematic review

## Abstract

**Background:**

The understanding of the prevalence and early predictive factors of scoliosis in children and adolescents is limited, which poses challenges to developing preventative strategies. This systematic review and meta-analysis aimed to clarify the prevalence and predictors of scoliosis among children and adolescents.

**Methods:**

We conducted a comprehensive search in PubMed, Cochrane, Embase, and Web of Science through October 2023. The quality of included studies was evaluated using the Joanna Briggs Institute scale or the Newcastle-Ottawa Scale. Subgroup analyses were performed to examine different types of scoliosis and specific demographic groups.

**Results:**

From 32 studies encompassing 55,635,351 children and adolescents, we identified 284,114 cases of scoliosis, resulting in a prevalence rate of 3.1% (95% CI: 1.5%–5.2%). This rate varied by gender, degrees of scoliosis severity, and between idiopathic vs. congenital forms. Notable predictors included gender, age, Body Mass Index (BMI), race, environmental factors, and lifestyle choices.

**Conclusion:**

Scoliosis is a significant condition affecting a minority of children and adolescents, particularly adolescent girls and individuals who are overweight. It is recommended that guardians and schools enhance educational efforts towards its prevention.

**Systematic Review Registration:**

https://www.crd.york.ac.uk/, Identifier CRD42023476498.

## 1 Introduction

Scoliosis is a three-dimensional (3D) spinal deformation characterized by a lateral curvature across one or more segments of the spine, coupled with vertebral rotation, resulting in core deviation and sagittal progression (1). Severe scoliosis can result in significant health complications, such as cardiovascular issues, reduced pulmonary function, chronic pain, and psychological distress (2).

Scoliosis can be categorized etiologically into idiopathic, congenital, and neuromuscular types. Adolescent idiopathic scoliosis (AIS), the most common form, has an unknown cause, which is reflected in the term “idiopathic” (3, 4). Depending on when it manifests, idiopathic scoliosis may be classified by age brackets: Infantile (ages 0–3 years), Juvenile (ages 3–10 years), Adolescent (ages 10–18 years), and Adult (ages above 18 years) (5). AIS is typically detected during childhood and adolescence, especially during rapid growth periods. Treatment options include observation, wearing orthotic braces, and surgery in more severe cases. Congenital scoliosis is caused by abnormal development of the spine during the embryonic stage, which may involve vertebral non-segmentation, abnormal shape, or abnormal quantity. This type of scoliosis is often associated with genetic factors and may coexist with other congenital abnormalities, so it is usually identified at birth or in early infancy (6). Treatment methods primarily involve surgical correction, especially in cases of severe or rapidly progressing curvature.

Currently, there is limited research available regarding the worldwide prevalence of scoliosis in children and adolescents, and a comprehensive investigation of associated risk factors is lacking. Consequently, the development of preventive strategies for children and adolescents faces considerable challenges. Therefore, the objective of this systematic review and meta-analysis was to provide a detailed description of the prevalence and early predictors of scoliosis in adolescents globally, and to offer evidence-based guidance for the detection and prevention of scoliosis.

## 2 Methods

### 2.1 Study registration

This meta-analysis was conducted in adherence to the Preferred Reporting Items for Systematic Reviews and Meta-Analyses (The PRISMA 2020) guidelines, and our protocol was registered with PROSPERO (ID: CRD42023476498).

### 2.2 Eligibility criteria

For our systematic review, we strictly included cohort or cross-sectional studies, while expressly excluding the following studies: (1) Studies that were based solely on subject self-reporting of disease diagnosis or assessments derived from specific scales that lacked clinical validation; (2) Case reports, meta-analyses, reviews, and guidelines; (3) Studies that had a sample size of fewer than 20 cases.

### 2.3 Data sources and search strategy

We systematically searched databases including PubMed, Cochrane Library, Embase, and Web of Science. The search was conducted from the inception of each database up to October 2023, without geographical restrictions. Details of the search are presented in Supplementary Table S1.

### 2.4 Study selection

All identified literature was imported into EndNote, where duplicate publications were initially filtered out automatically and manually. Studies relevant to our topic were selected by reviewing titles and abstracts, and then the full-texts of potentially relevant articles were downloaded and read. Finally, original research articles that met the inclusion criteria were selected upon full-text review. The literature screening process was independently carried out by two researchers. In the event of any discrepancies, a third researcher was consulted to discuss and make a final decision.

### 2.5 Data extraction

Before initiating data extraction, we devised a standardized template to systematically collect relevant data. The details gathered included DOI/PMID, first author, year of publication, type of study, author's nationality, patient demographics, period of sample collection, age group included, scoliosis categories, scoliosis diagnostic criteria, total participant count, number of scoliosis cases, along with age, gender, and independent correlation factors.

Data extraction was independently carried out by two reviewers. In instances of disagreement, a third reviewer was consulted to contribute to the resolution process.

### 2.6 Risk of bias in studies

The original studies encompassed in this systematic review were either cohort or cross-sectional studies. For the cohort studies, we evaluated their quality using the Newcastle-Ottawa Scale (NOS), a renowned assessment tool designed to judge the quality of non-randomized studies in meta-analyses (7). The NOS examines three domains through eight items, allocating up to one point each for most questions, with the exception of the comparability category, which has a potential for 2 points. Studies achieving a score between 7 and 9 are considered high quality, whereas scores from 4 to 6 signify moderate quality. For cross-sectional studies, the appraisal of quality was conducted using the Joanna Briggs Institute (JBI) scale (8).

The risk of bias in these studies was assessed independently by two researchers. In cases of discrepancy, a third researcher was enlisted to reach a consensus.

### 2.7 Synthesis methods

Data analysis for this meta-analysis was conducted using R software (version 4.2.2). Prior to performing the meta-analysis of prevalence, data underwent transformation based on predefined criteria: (1) No transformation was necessary if the average prevalence rate across all samples was between 20% and 80%; (2) Logit transformation was applied when the rate was under 20% or exceeds 80%; and (3) The double arcsine transformation method was applied in cases with a significant number of extreme values (0% or/and 100%) (9). The choice of model was guided by the level of heterogeneity, which was determined by the *I*^2^ index. A random-effects model was employed when *I*^2^ exceeded 50%; otherwise, a fixed-effect model was used. In circumstances of substantial heterogeneity, sensitivity and subgroup analyses were conducted to investigate potential sources of variability. Funnel plots were constructed to visually inspect for publication bias across the studies, complemented by statistical assessment via Egger's test. In instances where publication bias was detected, the trim-and-fill method was applied to evaluate its influence on the meta-analysis outcomes. A *P*-value of less than 0.05 was considered indicative of statistical significance.

## 3 Results

### 3.1 Study selection

**Figure 1:**
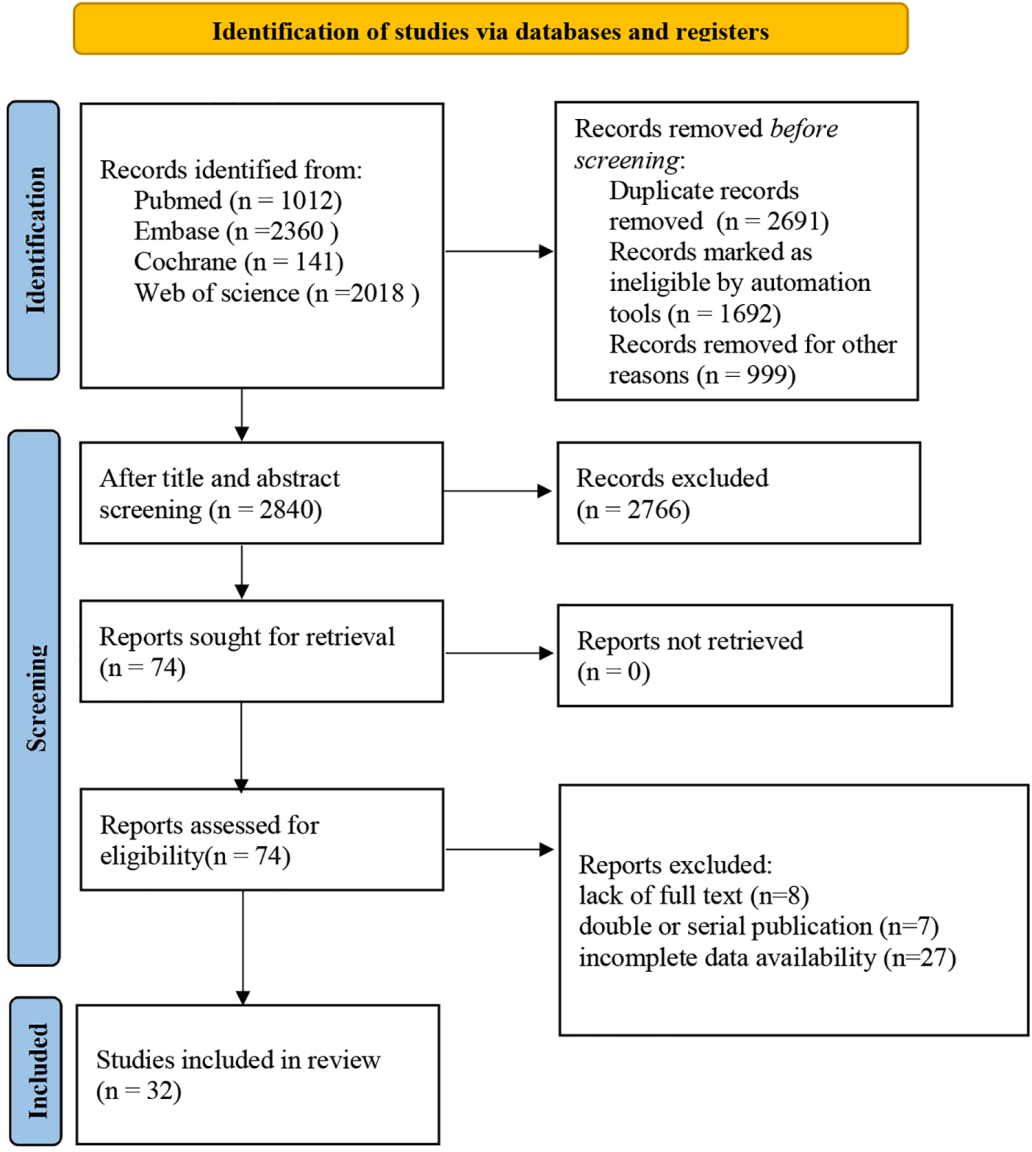
Literature screening process.

We retrieved a total of 5,531 records. Following the elimination of duplicates, 2,840 records were screened by their titles and abstracts. Of these, 2,766 records were excluded for not meeting our inclusion criteria. A thorough assessment of the full texts for the remaining 74 articles resulted in further exclusion of 42 articles for various reasons including unavailability of full text (*n* = 8), duplicate or serial publications (*n* = 7), and incomplete data (*n* = 27). Consequently, 32 studies were included in our meta-analysis (8–39) (Figure 1).

### 3.2 Study characteristics

This analysis incorporated 32 epidemiological investigations conducted from 1982 to 2022, involving a total of 55,635,351 children and adolescents. Among these, there were 29 cross-sectional studies (8–17, 19–21, 23–26, 39) and 3 cohort studies (18, 22, 29). The research spanned across different regions, with 21 studies (9, 12, 15–17, 23–29, 31–33, 39) conducted in Asia, including China, South Korea, Thailand, Japan, India, Singapore, Indonesia, and Iran; 6 studies (8, 10, 13, 14, 19, 30) in Europe from countries such as Sweden, England, Bosnia and Herzegovina, Portugal, and Turkey; 3 studies (11, 20, 21) in Brazil in South America; and 2 studies (18, 22) in the United States in North America. Most participants were 10–15 years old. All studies shared a common standard for diagnosing scoliosis: a positive Adam's Forward Bending Test (FBT) and a Cobb angle greater than 10°. Of these studies, 21 (19–33, 39) reported on the prevalence of idiopathic scoliosis, 5 (17, 18, 24, 31, 36) on congenital scoliosis, and the remaining 9 studies (8–16) did not specify the type of scoliosis (Table 1).

**Table 1:** Characteristics of the included studies.

No.	First author	Publication period	Type of design	Author's country	Patient origin	Sampling time	The age stage of the included population	Types of scoliosis	Number of scoliosis people	Total number of people	Age	Gender (M/F)
1	Yan Zou	2022	Cross-sectional study	China	The sampling covers all prefecture-level cities in Zhejiang Province. Based on the whole class, at least 80 students in each grade of primary school, junior high school, and senior high school were selected.	2019	6–17	Idiopathic scoliosis	1,766	45,547	NR	23,706/21,841
2	Sahyun Sung	2021	Cohort study	Korea	The universal health coverage system of South Korea, the National Health Insurance (NHI)	2011.1.1–2015.12.31	0–18	Idiopathic scoliosis	267,283	53,773,663	13.56 ± 3.35	28,024,720/25,748,943
3	Lijin Zhou	2022	Cross-sectional study	China	In Qinghai-Tibetan Plateau from schools and nearby villages	2020.5–2020.12	6–17	Idiopathic scoliosis, Congenital scoliosis, Neuromuscular scoliosis, Syndromic scoliosis, Scoliosis	364	9,856	NR	5076/4780
4	Yu Zheng	2016	Cross-sectional study	China	Based on a representative sample from Beitang District, Wuxi.	March–June 2014	6–13	Idiopathic scoliosis	11	11,024	10.21 ± 1.89	5,908/5,116
5	Flordeliza Yong,	2009	Cross-sectional study	Singapore	Singapore primary schools and secondary schools	2003	9–13	Idiopathic Scoliosis	1,118	93,626	NR	93,626 female
6	Hurriyet Yılmaz	2020	Cross-sectional study	Turkey	Children aged 10–15 years in Turkey.	2016	10–15	Idiopathic Scoliosis	369	16,045	12.0 ± 1.3	7,883/8,162
7	Hee-Kit Wong	2005	Cross-sectional study	Singapore	Randomly selected schools in Singapore	1997	6–14	Idiopathic Scoliosis	429	72699	NR	35,558/37,141
8	Stig Willner	1982	Cross-sectional study	Sweden	In Malmo, Sweden, 17,181 school children born in the years 1961–1965 were screened for scoliosis once a year between the ages of 7 and 16 years, during 197 1–1980.	1971–1980.	7–16	Scoliosis	474	17,181	NR	8,712/8,469
9	Fei Wang	2021	Cross-sectional study	China	A tertiary children's hospital	February 2008 and September 2019	0–3	Congenital scoliosis	89	50426	(70 ± 98 days)	31,072/19,354
10	Masaki Ueno	2011	Cross-sectional study	Japan	School	2003 and 2007	11–14	Idiopathic scoliosis	2,225	255,875	NR	127,972/127,903
11	J. STIRLING	1996	Cross-sectional study	England	Sixty-two schools in the Leeds region		6–14	Idiopathic Scoliosis	76	15,799	NR	NR
12	Hemender Singh	2022	Cross-sectional study	India	Different educational institutions of Jammu region in Jammu and Kashmir		10–28	Idiopathic scoliosis, Congenital scoliosis, Infantile Scoliosis, Kyphoscoliosis Scoliosis, Functional Scoliosis, Scoliosis	58	9,500	NR	5,001/4,499
13	Comron Saifi	2012	Cohort study	America	NR	Between 1992 and 2007	NR	Congenital scoliosis	12	364	NR	216/148
14	Patrícia Jundi Penha	2018	Cross-sectional study	Brazil	The data used in this study were collected at public schools in three cities within the state of São Paulo: Amparo, Pedreira, and Mogi Mirim.		10–14	Idiopathic scoliosis	37	2,562	NR	1,072/1490
15	Zdenko Ostoji	2005	Cross-sectional study	Bosnia and Herzegovina	Bosnia and Herzegovina.	In the school-year 2002/2003	7–14	Scoliosis	79	2,517	NR	1,272/1245
16	Lenice Sberse Nery	2010	Cross-sectional study	Brazil	In schools in the municipality of Carlos Barbosa, Rio Grande do Sul.	This study was carried out in February,March, April and May 2008	10–14	Scoliosis	19	1,340	NR	684/656
17	Sepehr Moalej	2018	Cross-sectional study	Iran	Primary schools in the 17th district of Tehran	October 2016 to February 2017.	7–12	Scoliosis	2	144	9.5 ± 1.71.	72/72
18	Beatriz Minghelli	2014	Cross-sectional study	Portugal	In public schools from all municipalities of the Algarve		10–16	Scoliosis	41	966	12.24 ± 1.53	437/529
19	Jin-Young Lee	2014	Cross-sectional study	Korea	In Ulsan	2004–2006	11	Idiopathic scoliosis	71	37,856	11.5 ± 0.3	20,746/17110
20	Sombat Kunakornsawat MD	2017	Cross-sectional study	Thailand	Randomly selected 10 out of 37 primary schools located in the Bangkok primary education area.		11–13	Idiopathic scoliosis	84	1,818	NR	NR
21	Zahed Safikhani	2006	Cross-sectional study	Iran	Secondary schools in Ahwaz City, Southwestern Iran	2004	11–15	Scoliosis	28	1,400	NR	NR
22	Komang-Agung IS	2017	Cross-sectional study	Indonesia	In Surabaya	2010	9–16	Idiopathic scoliosis	23	784	NR	315/496
23	Janani	2019	Cross-sectional study	India	Thiruvallur district	November 2016–August 2017.	11–15	Idiopathic scoliosis	164	3,250	NR	1,685/1,565
24	Fuli Huang	2019	Cross-sectional study	China	Junior high school students in Zhongshan city	July 2015–December 2017	11–15	Idiopathic scoliosis, Congenital scoliosis	646	41,258	13.3 ± 2.4	21,342/19,916
25	Miao Hu	2022	Cross-sectional study	China	In the Huangpu district, Shanghai, China. All 6th to 8th grade students in Huangpu district	2019	10–15	Idiopathic scoliosis	214	10,731	13.13 ± 0.93	5,518/5,213
26	Fan Hengwei	2016	Cross-sectional study	China	In Guangdong province.	September 2013 and July 2014	10–19	Idiopathic scoliosis	5,125	99,695	NR	50,538/49,157
27	Mohammadreza Etemadifar	2020	Cross-sectional study	Iran	24 schools were randomly chosen from six zones	November 2014–March 2015	10–14	Idiopathic scoliosis	19	3,018	12.26 ± 1.48	1,505/1,513
28	Murat Şakir Ekşi	2019	Cross-sectional study	Turkey	A community-based hospital located	NR	12–17	Scoliosis	111	1,065	14.95 ± 1.14	546/519
29	Qing Du	2014	Cross-sectional study	China	The Chongming Ministries of Education and Health, each school was contacted.	From April through November 2012	6–17	Scoliosis	172	6,824	NR	3,477/3347
30	J. S. DARUWALLA	1985	Cross-sectional study	Singapore	NR	1982	6–17	Scoliosis	1,096	110744	NR	50,577/60,167
31	Milla Gabriela Belarmino Dantas	2021	Cross-sectional study	Brazil	8 public schools in 2 cities from the semiarid region of Pernambuco	April and December 2017, April 2018 and June 2019	10–16	Idiopathic scoliosis	16	520	NR	221/299
32	Kevin Bondar, B.S	2021	Cohort study	America	The Kaiser Permanente health system	January 1, 2013 until the end of 2013	0–17	Idiopathic scoliosis	1,893	937,254	NR	478,611/456,750

### 3.3 Risk of bias in studies

Due to the inclusion of both cross-sectional and cohort studies, we utilized the NOS and JBI to assess their quality, respectively. The NOS revealed that the three cohort studies scored between 7 and 9, indicating that they are of high quality. The JBI demonstrated that the cross-sectional studies had no significant risk of bias (Supplementary Table S2).

### 3.4 Meta-analysis

#### 3.4.1 Scoliosis

(1) Synthesized results

**Figure 2:**
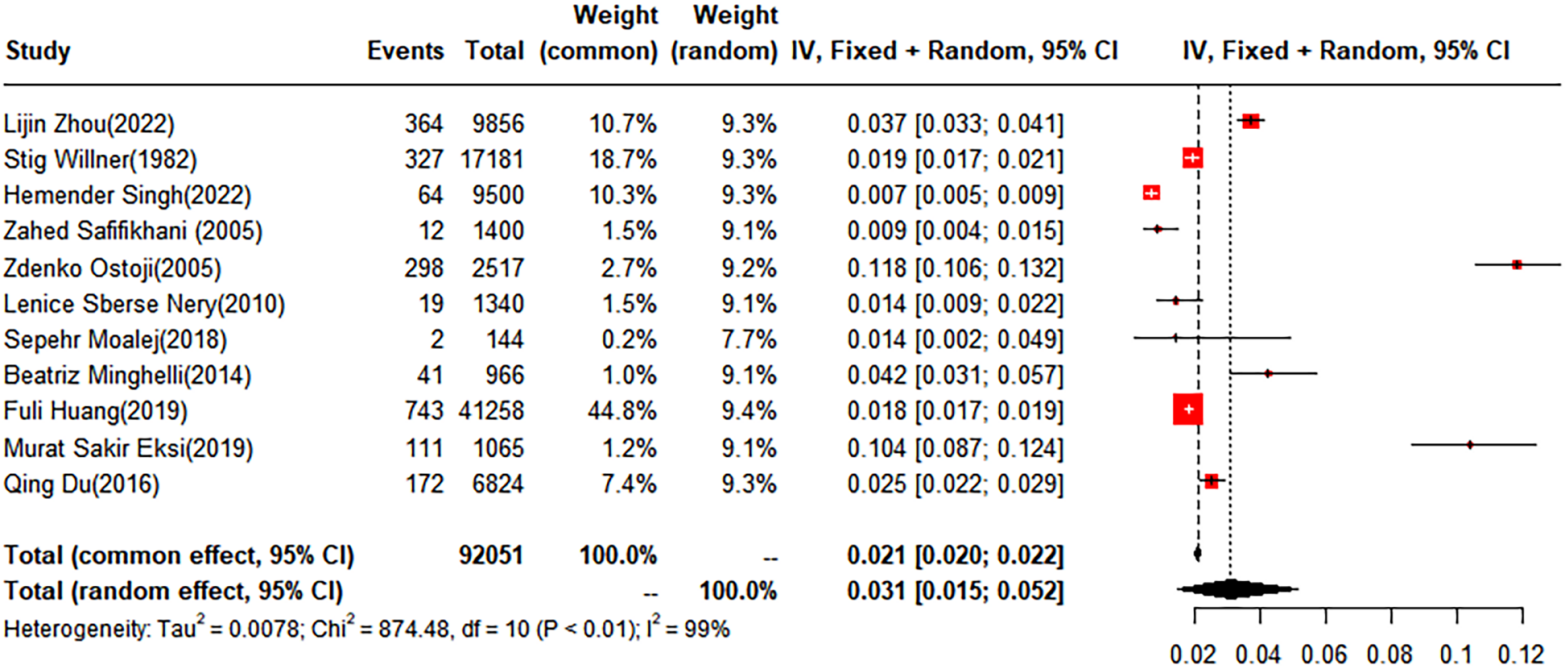
Forest plot for the meta-analysis of the prevalence of scoliosis in children and adolescents.

In this analysis, 11 studies did not categorize scoliosis. A random-effects model was used for data analysis, and the pooled prevalence rate of scoliosis was found to be 3.1% (95% CI: 1.5%–5.2%) (Figure 2).

(2) Subgroup analysis

The pooled prevalence of scoliosis was found to be 2.58% (95% CI: 1.11%–4.62%) in males and 4.06% (95% CI: 1.96%–6.48%) in females. When classified by degree, the prevalence rates for scoliosis within the subgroups of 10–19 degrees, 20–29°, and over 40° were 3.01% (95% CI: 0.67%–6.94%), 0.36% (95% CI: 0.32%–0.41%), and 0.05% (95% CI: 0%–0.18%), respectively. Geographically, the children and adolescents included in the study were from four regions: Asia, Europe, South America, and North America. The prevalence rates for scoliosis in the Asian and European groups were 1.70% (95% CI: 0.88%–2.77%) and 6.42% (95% CI: 2.42%–12.13%), respectively. No studies conducted in North America met our inclusion criteria. Of the included studies, five conducted a subgroup analysis based on anatomical location, dividing cases into thoracic, thoracolumbar, and lumbar. The prevalence rates for the thoracic and thoracolumbar groups were 3.89% (95% CI: 0%–14.71%) and 1.18% (95% CI: 0.38%–2.39%), respectively (Table 2).

(3) Sensitivity analysis and publication bias

**Table 2:** Subgroup analysis results for prevalence of scoliosis in children and adolescents.

Subgroup	Value	Literature quantity	Number of scoliosis cases	Total cases	ES (95% CI)	*I*^2^ (%)
Site	Thoracic	2	160	7,889	3.89% (0.00%–14.71%)	99.30
	Thoracolumbar	2	72	7,889	1.18% (0.38%–2.39%)	87.70
	Lumbar	1	34	6,824	NA	NA
Degree	10°–19°	4	1,066	66,328	3.01% (0.67%–6.94%)	98.50
	20°–29°	2	213	58,439	0.36% (0.32%–0.41%)	0.00
	30°–39°	1	31	17,181	0.18% (0.12%–0.25%)	NA
	>40°	2	24	58,439	0.05% (0.00%–0.18%)	95.00
District	Asia	6	1,357	68,982	1.70% (0.88%–2.77%)	98.10
	Europe	4	777	21,729	6.42% (2.42%–12.13%)	99.40
	South America	1	19	1,340	NA	NA
Sex	Boy	10	762	46,619	2.58% (1.11%–4.62%)	97.70
	Girl	10	1,379	44,032	4.06% (1.96%–6.84%)	98.50

**Figure 3:**
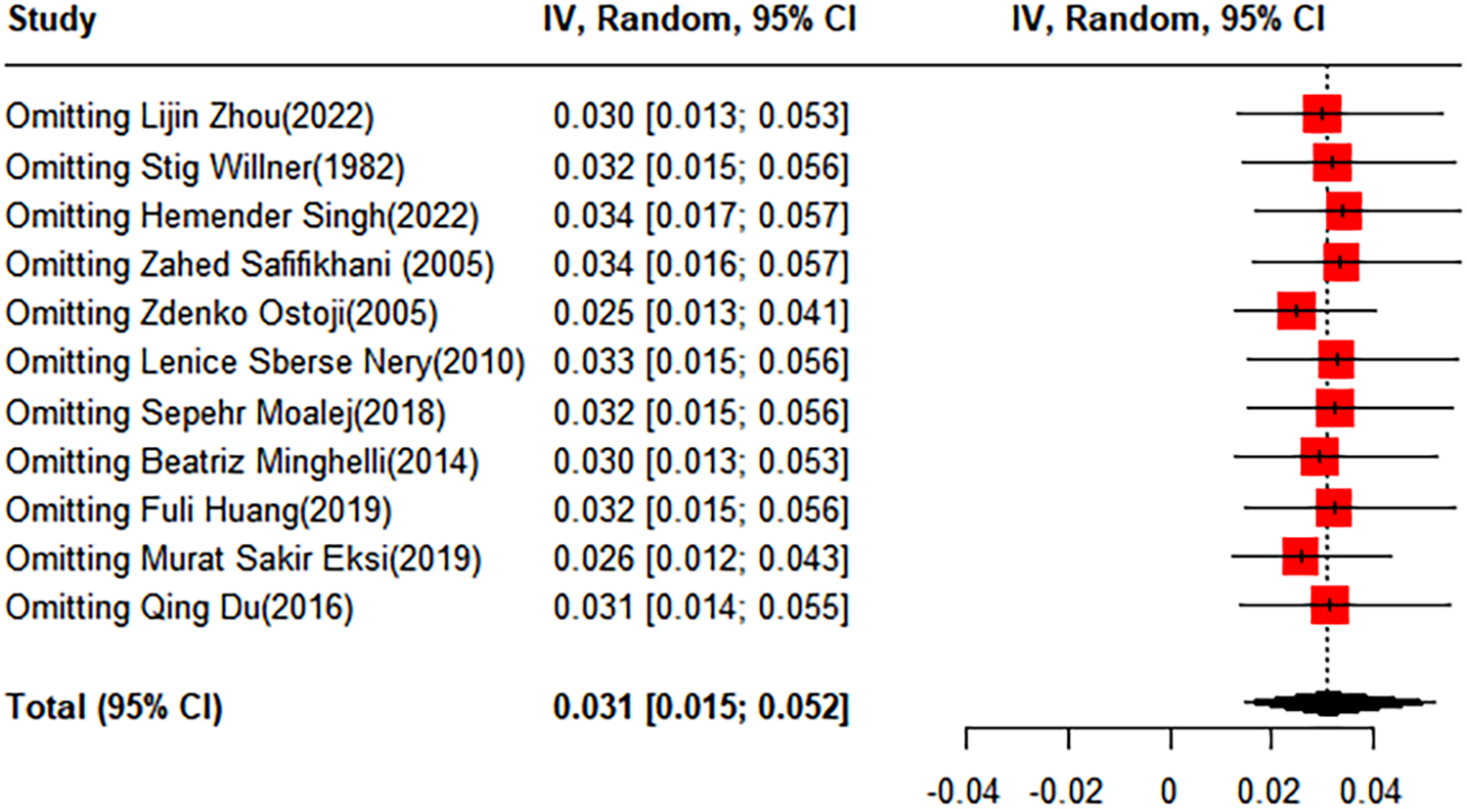
Forest plot for the sensitivity analysis of the prevalence of scoliosis in children and adolescents.

**Figure 4:**
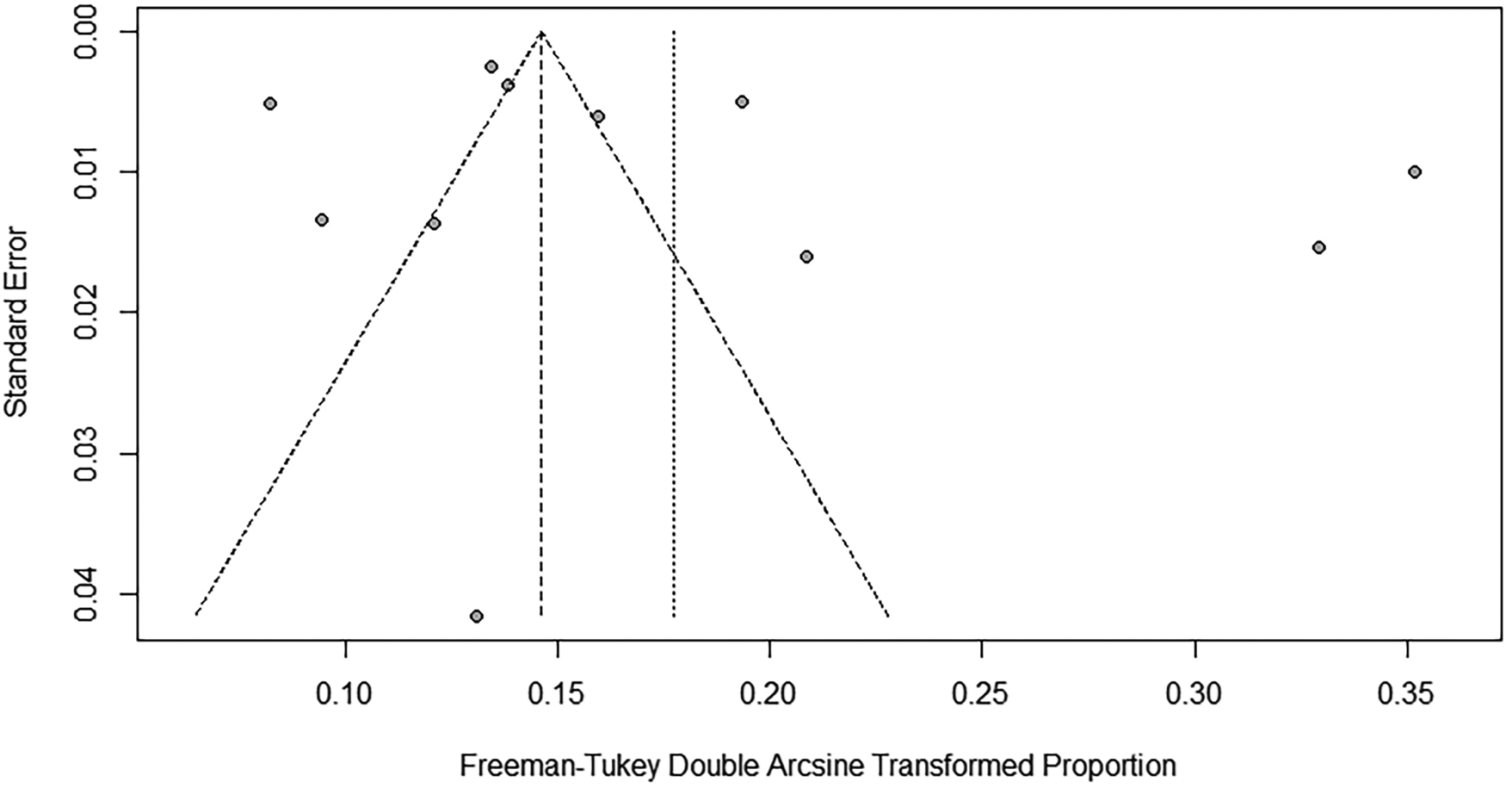
Funnel plot for publication bias in prevalence of scoliosis in children and adolescents.

To evaluate the robustness and reliability of the overall findings of the meta-analysis, a sensitivity analysis was performed. This technique involved sequentially omitting individual studies and recalculating the meta-analysis with the remaining studies. We investigated whether this exclusion led to significant deviations in the outcomes, thereby confirming the sturdiness of our conclusions. Funnel plots were used to assess potential publication bias, and the results indicated the presence of publication bias. Consequently, the trim-and-fill method was implemented. When two studies were added, the result was adjusted to 1.9% (95% CI: 0.49%–4.15%) (Figures 3, 4).

#### 3.4.2 Idiopathic scoliosis

(1) Synthesized results

**Figure 5:**
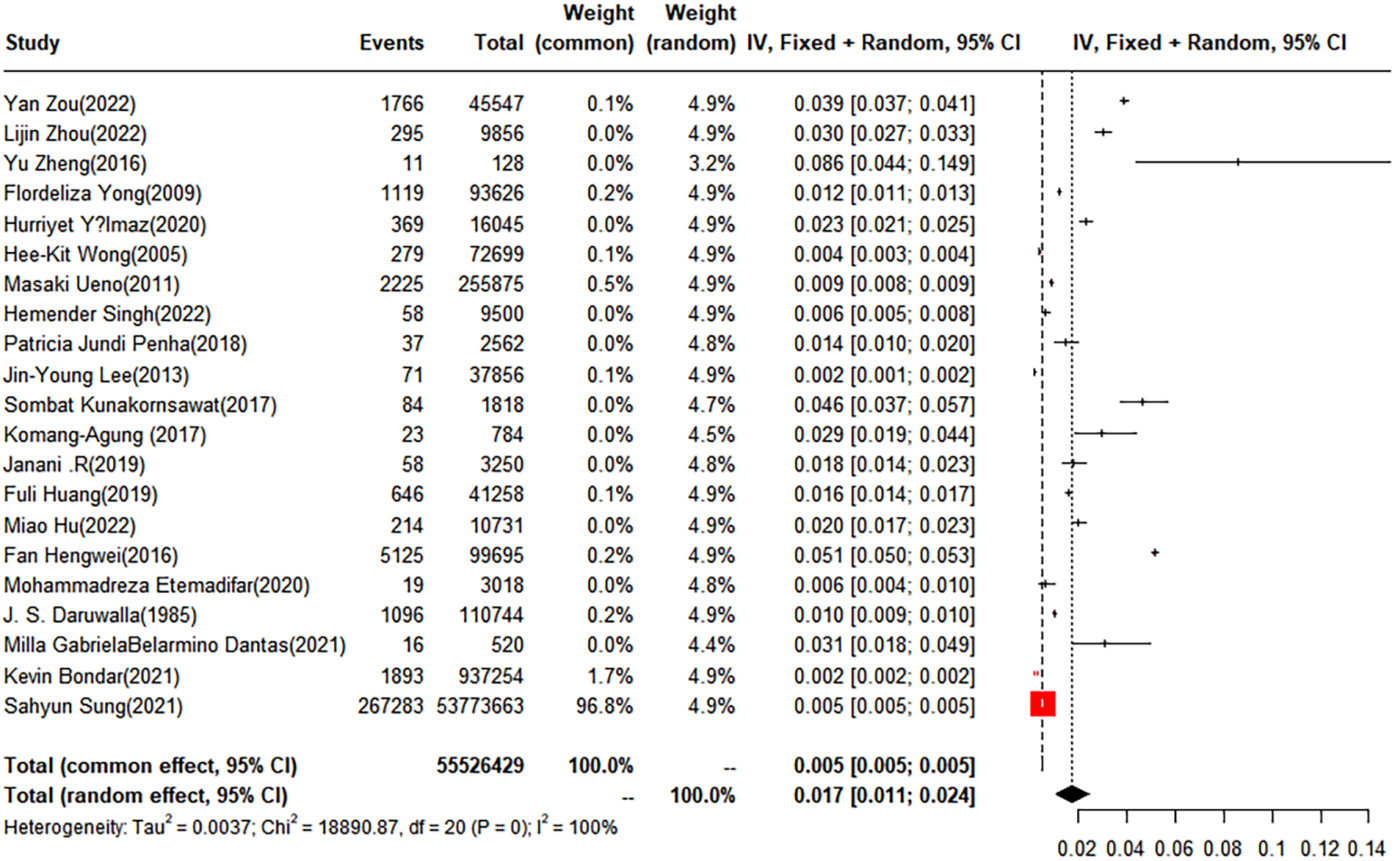
Forest plot for the meta-analysis of the prevalence of idiopathic scoliosis in children and adolescents.

Out of the studies reviewed, 21 reported the prevalence of idiopathic scoliosis. A random-effects model was used, and the calculated prevalence rate for idiopathic scoliosis was found to be 1.7% (95% CI: 1.1%–2.4%) (Figure 5).

(2) Subgroup analysis

The pooled prevalence was 1.12% (95% CI: 0.55%–1.89%) for males and 4.51% (95% CI: 0.74%–11.09%) for females. Subgroup analysis by degree unraveled that the prevalence of idiopathic scoliosis in subgroups with curves of 10–19°, 20–29°, 30–39°, and greater than 40° was 1.46% (95% CI, 0.64–2.58), 0.35% (95% CI, 0.08–0.8), 0.07% (95% CI: 0%–0.21%), and 0.19% (95% CI: 0.03%–0.48%), respectively. Geographically, the included children and adolescents were divided into four groups: Asia, Europe, South America, and North America. The prevalence of idiopathic scoliosis was 1.68% (95% CI: 0.94%–2.63%) in Asia, 1.22% (95% CI: 0.09%–3.64%) in Europe, and 2.08% (95% CI: 0.76%–4.01%) in South America. One study conducted in North America was not suitable for subgroup analysis. Five studies provided a subgroup analysis by anatomical site, categorizing the cases as thoracic, thoracolumbar, and lumbar. The prevalence rates for scoliosis within these groups were 0.44% (95% CI: 0.15%–0.88%), 0.44% (95% CI: 0.19%–0.77%), and 0.16% (95% CI: 0.02%–0.42%), respectively. Based on the developmental status of the children and adolescents, the analysis was further subdivided into three groups: normal weight, overweight, and obesity, with the prevalence rates being 1.84% (95% CI: 0%–7.44%), 1.29% (95% CI: 0%–5.80%), and 0.77% (95% CI: 0%–4.48%), respectively. In addition to these categories, we conducted a subgroup analysis based on the number of curves. The results showed that the prevalence of scoliosis stood at 0.68% (95% CI: 0.11%–1.73%) for single curve, 0.31% (95% CI: 0.03%–0.90%) for double curve, and 0.05% (95% CI: 0.03% - 0.06%) for triple curves. A subgroup analysis based on race was also performed, involving four groups: White, Black, Yellow, and Other. Two studies reported a prevalence of 0.83% (95% CI: 0%–3.20%) in the White population, and two studies reported a prevalence of 0.28% (95% CI: 0%–1.16%) in the Black population. The Yellow and Other groups each had only one study reporting prevalence; therefore, no subgroup analysis was performed (Table 3).

(3) Sensitivity analysis and reporting biases

**Table 3:** Subgroup analysis results for prevalence of idiopathic scoliosis in children and adolescents.

Subgroup	Value	Literature quantity	Number of scoliosis cases	Total cases	ES (95% CI)	*I*^2^ (%)
Site	Thoracic	7	437	177,894	0.44% (0.15%; 0.88%)	97.80
	Thoracolumbar	7	637	177,895	0.44% (0.19%; 0.77%)	96.80
	Lumbar	7	132	177,896	0.16% (0.02%; 0.42%)	97.00
Degree	10°–19°	8	6,723	532,229	1.46% (0.64%; 2.58%)	99.90
	20°–29°	5	1,325	249,040	0.35% (0.08%; 0.80%)	99.60
	30°–39°	4	329	249,040	0.07% (0.00; 0.21%)	94.70
	>40°	6	135	1,241,030	0.19% (0.03%; 0.48%)	99.80
	>20°	2	808	271,674	0.17% (0.02%; 0.49%)	98.00
District	Asia	16	279,600	54,470,200	1.68% (0.94%; 2.63%)	99.90
	Europe	2	445	31,844	1.22% (0.09%; 3.64%)	99.50
	North America	1	1,893	937,254	NA	NA
	South America	2	53	3,082	2.08% (0.76%; 4.01%)	82.60
Sex	Boy	19	118,652	28,723,827	1.12% (0.55%; 1.89%)	99.70
	Girl	19	162,262	26,430,274	4.51% (0.74%; 11.09%)	99.90
Develop	Missing	1	30	231,399	NA	NA
	Underweight	1	61	11,504	NA	NA
	Normal weight	2	2,663	360,650	1.84% (0.00; 7.44%)	100.00
	Overweight	2	459	115,277	1.29% (0.00; 5.80%)	99.80
	Obesity	2	227	89,928	0.77% (0.00; 3.48%)	99.50
Number	Single curve	3	943	147,527	0.68% (0.11%; 1.73%)	99.50
	Double curve	3	562	147,527	0.31% (0.03%; 0.90%)	99.40
	Triple curves	2	53	109,671	0.05% (0.03%; 0.06%)	0.00
Race	White	2	550	226,476	0.83% (0.00; 3.20%)	97.90
	Black	2	139	80,127	0.28% (0.00; 1.16%)	68.90
	Yellow	1	–	4	NA	NA
	Other	1	7	791	NA	NA

**Figure 6:**
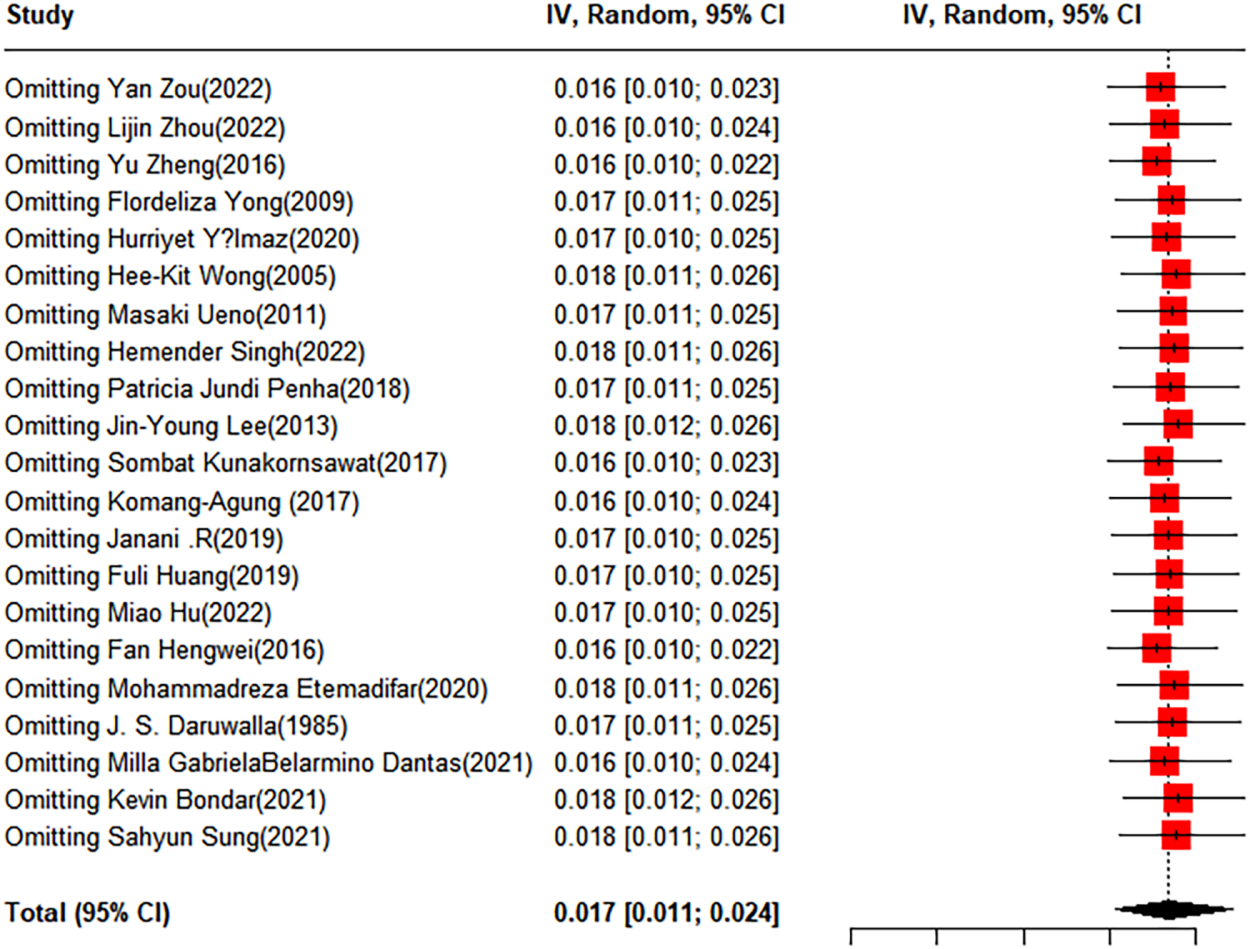
Forest plot for the sensitivity analysis of the prevalence of idiopathic scoliosis in children and adolescents.

**Figure 7:**
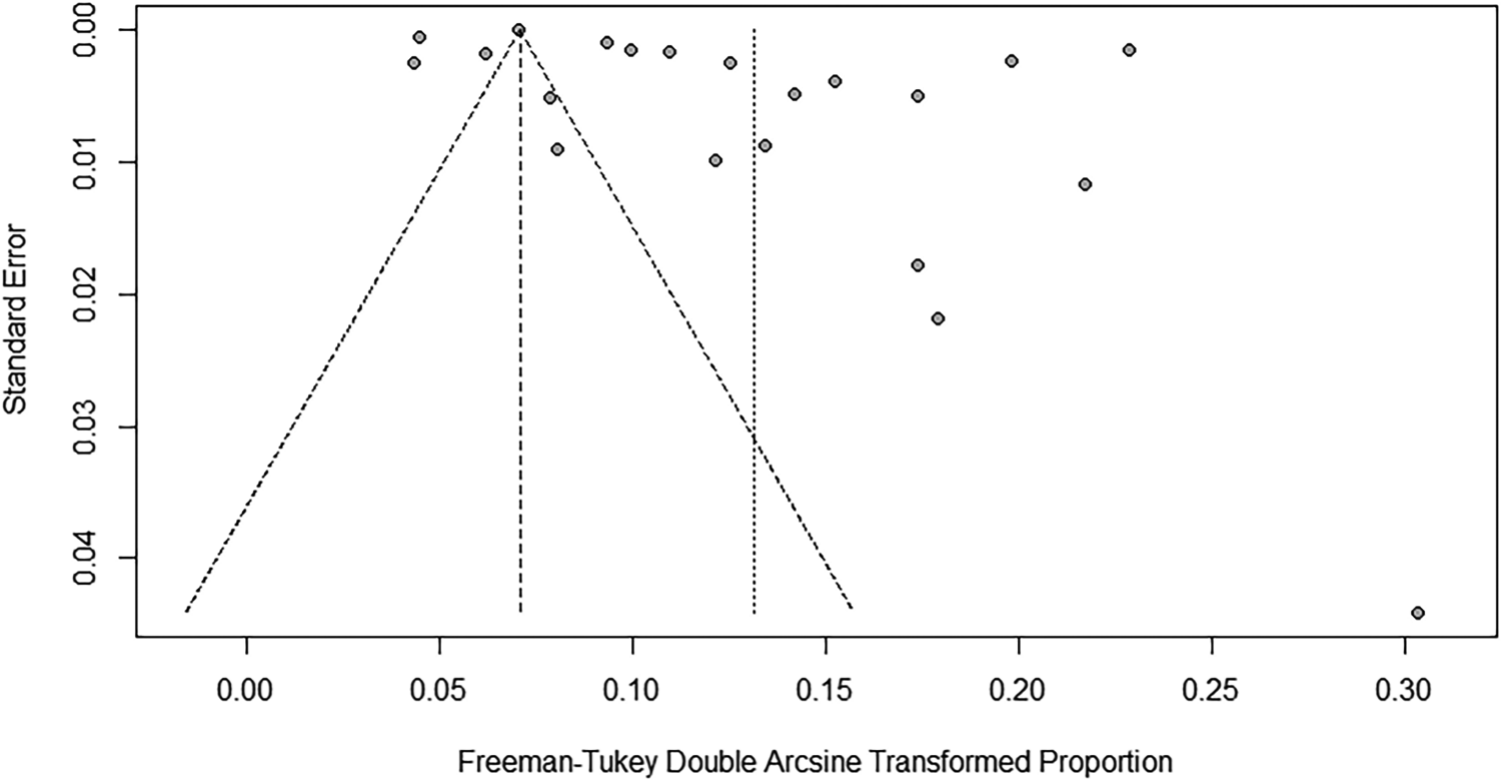
Funnel plot for publication bias regarding the prevalence of idiopathic scoliosis in children and adolescents.

To evaluate the robustness and reliability of the overall results of the meta-analysis, a sensitivity analysis was conducted. This method entailed sequentially excluding individual studies and performing a meta-analysis with the remaining data to determine whether the recalculated results were significantly different from the initial results, thereby affirming the solidity of our conclusions. The funnel plot results indicated the existence of publication bias; hence, we applied the trim-and-fill method, introducing 10 additional studies into the analysis. After this adjustment, the prevalence rate was recalculated as 0.53% (95% CI: 0.12%–1.2%) (Figures 6, 7).

#### 3.4.3 Congenital scoliosis

Congenital scoliosis is a type of spinal deformity present at birth, associated with anomalies in the spine's development during the embryonic stage. This condition is distinct from AIS, which typically occurs during puberty and the cause is unknown. Congenital scoliosis can be caused by vertebral malformations, fusion of the vertebrae, or other spinal abnormalities. The incidence of congenital scoliosis is much lower than that of AIS. In this meta-analysis, four studies discussed congenital scoliosis, covering 101,548 children and adolescents, among whom 218 cases of congenital scoliosis were reported. The estimated prevalence rate of congenital scoliosi was approximately 0.215%.

#### 3.4.4 Independent risk factors

Seven studies reported independent risk factors, with substantial heterogeneity observed among them. We identified gender, age, abnormal body mass index (BMI) (either underweight or overweight), racial differences, environmental factors, and lifestyle factors (such as prolonged sitting, lack of exercise, insufficient sleep) as independent risk factors. The use of single-strap bags and carrying overweight backpacks were also closely linked to the condition. Family income level, as an indicator of nutrition and medical conditions, was regarded as a potential risk factor. Detailed analyses can be found in Supplementary Table S3.

## 4 Discussion

### 4.1 Summary of the main findings

This study highlighted a relatively high prevalence of scoliosis among children and adolescents, with idiopathic scoliosis and congenital scoliosis warranting particular attention. The outcomes of our research indicated that the prevalence of scoliosis stood at 3.1% (95% CI: 1.5%–5.2%), idiopathic scoliosis at 1.7% (95% CI: 1.1%–2.4%), and congenital scoliosis at 0.215% (95% CI: 0.12%–1.2%).

In this study, we also discovered significant variations in the prevalence of scoliosis across different regions. The prevalence of scoliosis among children and adolescents was higher in Europe than in Asia. The incidence of AIS in South America was higher than that in Asia and Europe. The occurrence of scoliosis may be influenced by a multitude of factors including genetics, environment, and lifestyle. Typically, there are differences in these factors between Western countries and Asian nations, which could lead to varying prevalence of scoliosis across different regions. We found that the primary angle of scoliosis was 10°–19°, with a prevalence rate of 3.01%, and the number of cases decreased as the angle increased. Idiopathic scoliosis also occurred mostly at 10°–19°, and the number of affected individuals reduced as the angle became more acute. The prevalence of scoliosis in the thoracic region was 3.89%, higher than the prevalence of 1.18% in the thoracolumbar region. The prevalence rates of idiopathic scoliosis in the thoracic and thoracolumbar regions were both 0.44%, and the prevalence of lumbar scoliosis was 0.16%, meaning that in cases of idiopathic scoliosis, thoracic and thoracolumbar scoliosis tend to have higher prevalence rates.

### 4.2 The risk factors associated with scoliosis

Gender significantly impacts the prevalence and progression of scoliosis. Substantial research, including our study, have consistently indicated that females are disproportionally affected by scoliosis compared to males. This meta-analysis demonstrated that the prevalence of scoliosis is markedly higher in females, with a pooled prevalence rate of 4.06%, as opposed to 2.58% in males. This discrepancy is especially prominent during adolescence, a peak period for scoliosis onset. A primary factor behind this increased prevalence in females may be attributed to estrogen influences. During puberty, elevated estrogen levels in females may impact spinal column growth and development. A proposed mechanism by which estrogen contributes to AIS involves delayed menarche, leading to extended prolonged periods of rapid growth when the spine is particularly vulnerable to deformities. Further, low estrogen levels associated with delayed menarche could result in diminished bone mineralization and strength, thereby escalating the risk of spinal deformities (40). Additionally, polymorphisms in estrogen receptors (ER α and ER β) have been linked to AIS, potentially due to mutations that affect the expression of downstream genes critical for bone growth and metabolism. Evidence from several studies supports the connection between specific ER polymorphisms and the incidence of AIS, indicating a genetic predisposition moderated by estrogen signaling pathways (41–43).

Scoliosis displays variable prevalence and progression across different age brackets. Data from our research identifies adolescence as a peak period for AIS onset. According to subgroup analysis, the total scoliosis prevalence among adolescents aged 10–15 years stands at 3.01% (95% CI: 0.67%–6.94%), emphasizing the heightened risk during the rapid growth phases of adolescence. During adolescence, the body undergoes rapid skeletal growth and development, substantially increasing scoliosis incidence. The rapid growth might incite spinal instability, thereby predisposing the spine to curvature and deformation (44). While scoliosis predominantly occurs during adolescence, its presence in infancy and early childhood should not be overlooked. Infantile Idiopathic Scoliosis (IIS), for instance, involves growth suppression of the vertebral growth plates on the concavity of the deformity, leading to vertebral body wedging and spinal buckling (45). Early detection and intervention are crucial in curtailing further progression of these spinal deformities. Our study findings indicate a 0.215% overall prevalence of congenital scoliosis (95% CI: 0.12%–1.2%), underscoring the importance of routine spinal examinations during infancy.

The correlation between BMI and scoliosis is intricate, drawing considerable focus in epidemiological research. Scoliosis, a three-dimensional spinal deformity characterized by lateral curvature and vertebral rotation, may be influenced by various factors, including BMI. A Mendelian randomization analysis demonstrated a causal link between low BMI and AIS onset (46). Typically, a low BMI might reflect undernutrition or a frail physique, potentially compromising the development and support of bones and muscles, thus elevating spinal curvature risk (47). Conversely, a high BMI may increase the mechanical load on the spine, fostering structural alterations and instability. Furthermore, in overweight individuals, the distribution of body fat may influence spinal mechanics, contributing to scoliosis development (48). In a prevalence study involving 196 obese adolescents (mean BMI 36 kg/cm^2)^, the prevalence of AIS was found to be 12.2%, which is double the rate observed in the general population (49). Another cross-sectional study revealed a higher prevalence of obesity in patients with AIS compared to healthy controls (50).

Lifestyle factors significantly influence the development and progression of scoliosis. Sedentary behaviors, marked by prolonged sitting and minimal physical activity, have been linked to an increased risk of scoliosis. Inactivity may result in insufficient engagement of spinal muscles, leading to poor posture and muscular imbalances, which could contribute to the onset of scoliosis (51). A meta-analysis indicated that exercise interventions are notably more effective than conventional therapies in reducing Cobb's angle in adolescents with AIS. Among various exercise forms, yoga demonstrated the significant impact, reducing the Cobb's angle by an average of 4.60°, followed by core strength training, Physiotherapeutic Scoliosis-Specific Exercises (PSSE), Schroth exercises, and sling exercises. Alothough all exercise forms were effective, no significant differences were observed among them. These findings underscore the potential benefits of exercise-based treatments, particularly yoga, for managing AIS (52). Further insights from a Meta-analysis of randomized controlled trials have also demonstrated that Pilates exercise training effectively reduces Cobb's Angle, decreases trunk rotation, alleviates pain, enhances trunk motion, and improves the quality of life for individuals with scoliosis (53). This study emphasizes the importance of physical activity in lowering AIS prevalence and suggests that participation in organized sports may offer a protective effect, especially for girls. Notably, a lower prevalence of AIS was noted among pupils engaged in soccer (2.8%), handball (3.4%), and martial arts (3.9%), compared to those participating in swimming (8.6%), dancing (7.7%), and volleyball (8.2%). Additionally, a positive correlation was identified between the use of handheld electronic devices and the prevalence of scoliosis, indicating that increased screen time may be associated with a higher risk of AIS (54). A case-control study conducted in China revealed that poor reading and writing posture are linked to a heightened susceptibility to AIS. Moreover, heavy school bags are significantly associated with the development of AIS. Adolescents who engage in more than two hours of screen time during weekdays are at a greater risk for developing AIS. With respect to dietary habits, individuals who avoid milk and dairy products show a higher predisposition to AIS (55). Additionally, a cross-sectional study in Japan revealed a positive association between AIS risk and factors such as increased frequency, years of experience, and duration of ballet training. It also noted that participants with mothers affected by scoliosis exhibited elevated odds of developing AIS (56).

Melatonin, a hormone secreted by the pineal gland, plays a role in regulating circadian rhythms and sleep cycles. It also possesses antioxidant and immunomodulatory properties. Emerging research suggests melatonin's involvement in the pathogenesis of scoliosis, particularly AIS (57). A study indicates that individuals with scoliosis often exhibit reduced melatonin levels. This deficiency may interfere with normal spinal growth and development, thereby potentially increasing the risk of scoliosis (58). Furthermore, animal studies, including those on salmon and chickens with induced melatonin deficits, have demonstrated a higher incidence of scoliosis. Remarkably, melatonin supplementation in these models has shown potential in partially reversing the condition (59).

### 4.3 The prevention strategies for scoliosis

To effectively mitigate the risks associated with scoliosis, a comprehensive and structured prevention strategy is paramount. This strategy should include regular screening, public education, lifestyle modifications, nutritional interventions, ergonomic improvements, medical interventions, and psychosocial support.

Firstly, regular screening is critical for early detection. Implementing school-based screening programs and integrating spinal examinations into routine pediatric check-ups can facilitate early identification of scoliosis, particularly during adolescence, when the risk is most pronounced. Secondly, public education plays a vital role. Informing parents, guardians, and students about the indicators and consequences of scoliosis, and emphasizing the importance of early intervention through school programs and public awareness campaigns can foster timely medical consultations and interventions. Thirdly, lifestyle modifications are necessary to mitigate risk. Promoting physical activity, especially those that enhance core strength such as yoga, Pilates, and Schroth exercises, can prevent the onset of scoliosis. Additionally, reducing sedentary behaviors, limiting screen time, and encouraging regular movement breaks can improve posture and spinal health. Fourthly, nutritional interventions are crucial. Ensuring an intake rich in calcium, vitamin D, and other vital nutrients supports bone health. Maintaining a healthy BMI through appropriate diet and regular physical activity is important, especially for children. Fifthly, ergonomic adjustments in daily activities can prevent scoliosis. Using both straps of backpacks, minimizing load, and ensuring that school furniture like desks and chairs are of appropriate sizes can promote good posture and reduce the risk of developing spinal deformities. Sixthly, For those at elevated risk, specific medical actions might be beneficial. Children with a family history or genetic predispositions to scoliosis should consider genetic counseling and monitoring of hormonal levels. Orthotic braces can manage early signs of scoliosis and prevent its progression. Lastly, psychosocial support is essential for the overall well-being of children diagnosed with scoliosis. Providing psychological support can help manage issues related to body image concerns and anxiety, ensuring a holistic approach to care for children.

By integrating these multi-faceted and logically structured prevention strategies, we can reduce the prevalence and severity of scoliosis in children and adolescents, leading to improved long-term health outcomes. Regular follow-ups and personalized care plans tailored to individual risk factors are integral to an effective scoliosis prevention program.

Aside from scoliosis, other orthopedic conditions are also prevalent in the minor population, such as fractures, osteochondritis dissecans (OCD), and Fallen Arches. OCD is a condition affecting subchondral bone and articular cartilage (60). In a study conducted by Jeffrey I Kessler in 2010 (61), the prevalence of OCD in the 6–19 age group was 9.5 per 100,000 individuals, with the highest prevalence in the 12–19 age group at 11.2 per 100,000. Multivariate logistic regression analysis indicated that the risk of developing OCD in children aged 12–19 was 3.3 times that in children aged 6–11. For children and adolescents, these orthopedic conditions can significantly impact the quality of their life later on, and should be afforded ample attention.

### 4.4 Advantages and limitations of the study

This systematic review and meta-analysis provide an exhaustive global perspective on the prevalence and predictors of scoliosis prevalence in children and adolescents. Rigorous methodologies coupled with detailed subgroup analyses ensure the reliablility of our findings and highlight key predictors for targeted prevention strategies. These insights provide a robust framework for improving scoliosis prevention and management.

However, there are limitations to this research. Firstly, despite its comprehensive scope, our study predominantly includes data from Asia and Europe, with scant representation from North and South America. This geographic discrepancy may rimpede the universal applicability of our conclusions. Future research should strive to encompass more studies from these under-represented regions to augment our understanding of the prevalence and predictors of scoliosis in children and adolescents globally. Additionally, conducting region-specific analyses could help identify unique risk factors and facilitate tailored prevention strategies accordingly. Secondly, the scarcity of data on congenital scoliosis hampers our capacity to thoroughly understand its epidemiology and associated risk factors. More in-depth studies focusing on congenital scoliosis are needed to refine prevalence estimates and improve preventive and therapeutic approaches.

In future research, we should incorporate literature covering wider ethnicities and diverse samples to validate the risk factors for scoliosis, aiming to develop standard or customized follow-up and screening strategies.

## 5 Conclusion

Our study found that the prevalence of scoliosis among children and adolescents was relatively high, with the degree mostly concentrated at 10°–19°. Female and overweight children and adolescents were more prone to developing scoliosis. The etiology of scoliosis may be related to a variety of factors, including genetic and environmental aspects. It is important to focus on prevention and timely diagnosis of scoliosis, particularly during adolescence. Preventative measures include maintaining the correct posture, engaging in physical exercise, and avoiding prolonged static positions. It should be emphasized that although scoliosis usually does not progress to a severe condition, it needs to be taken seriously, and medical evaluation and appropriate treatment should be promptly sought upon the appearance of early signs.

## Data Availability

The original contributions presented in the study are included in the article/Supplementary Material, further inquiries can be directed to the corresponding author.

## Supplementary material

Supplementary Table S1 Literatur search strategy.

Supplementary Table S2 Joanna Briggs Institute scale.

Supplementary Table S3 Independent risk factors.
