# Supplementary material for: Prevalence of scoliosis in children and adolescents: a systematic review and meta-analysis
Source: Front Pediatr. 2024 Jul 23;12:1399049. doi: 10.3389/fped.2024.1399049 (PMC11300313; doi:10.3389/fped.2024.1399049)
Supplement: Supplementary Table S1 — Literatur search strategy. [file Table1.docx]

# Table S1 Literature search strategy

# 1.Pubmed

| Search number | Query | Results |
| --- | --- | --- |
| #1 | "Minors"[Mesh] | 2834 |
| #2 | (((((((((((((((((((Minor[Title/Abstract]) OR (child[Title/Abstract])) OR (children[Title/Abstract])) OR (juvenile[Title/Abstract])) OR (Childhood[Title/Abstract])) OR (adolescent[Title/Abstract])) OR (Adolescents[Title/Abstract])) OR (Adolescence[Title/Abstract])) OR (Teens[Title/Abstract])) OR (Teen[Title/Abstract])) OR (Teenagers[Title/Abstract])) OR (Teenager[Title/Abstract])) OR (Youth[Title/Abstract])) OR (Youths[Title/Abstract])) OR (Younger[Title/Abstract])) OR (Pediatrics[Title/Abstract])) OR (Pediatric[Title/Abstract])) OR (Infants[Title/Abstract])) OR (Infant[Title/Abstract])) OR (Infantile[Title/Abstract]) | 2880560 |
| #3 | #1 or #2 | 2881991 |
| #4 | Scoliosis"[Mesh] | 21059 |
| #5 | ((Scolioses[Title/Abstract]) OR (kyphoscoliosis[Title/Abstract])) OR (kyfoscoliosis[Title/Abstract]) | 2219 |
| #6 | #4 or #5 | 22203 |
| #7 | Prevalence"[Mesh] | 347011 |
| #8 | (((Incidence[Title/Abstract]) OR (Incidences[Title/Abstract])) OR (Epidemiology[Title/Abstract])) OR (Epidemiologies[Title/Abstract]) | 1169949 |
| #9 | #7 or #8 | 1462598 |
| #10 | #3 and #6 and #9 | 1012 |

2.Cochrane

| Search number | Query | Results |
| --- | --- | --- |
| #1 | MeSH descriptor: [Minors] explode all trees | 11 |
| #2 | (Minors):ti,ab,kw OR (Minor):ti,ab,kw OR (child):ti,ab,kw OR (children):ti,ab,kw OR (juvenile):ti,ab,kw | 202051 |
| #3 | (Childhood):ti,ab,kw OR (adolescent):ti,ab,kw OR (Adolescents):ti,ab,kw OR (Adolescence):ti,ab,kw OR (Teens):ti,ab,kw | 178271 |
| #4 | (Teen):ti,ab,kw OR (Teenagers):ti,ab,kw OR (Teenager):ti,ab,kw OR (Youth):ti,ab,kw OR (Youths):ti,ab,kw | 11893 |
| #5 | (Younger):ti,ab,kw OR (Pediatrics):ti,ab,kw OR (Pediatric):ti,ab,kw OR (Infants):ti,ab,kw OR (Infant):ti,ab,kw | 123086 |
| #6 | (Infantile):ti,ab,kw | 2662 |
| #7 | #1 or #2 or #3 or #4 or #5 or #6 | 362582 |
| #8 | MeSH descriptor: [Scoliosis] explode all trees | 667 |
| #9 | (Scoliosis):ti,ab,kw OR (Scolioses):ti,ab,kw OR (kyphoscoliosis):ti,ab,kw OR (kyfoscoliosis):ti,ab,kw | 1711 |
| #10 | #8 or #9 | 1711 |
| #11 | MeSH descriptor: [Prevalence] explode all trees | 8722 |
| #12 | (Prevalence):ti,ab,kw OR (Incidence):ti,ab,kw OR (Incidences):ti,ab,kw OR (Epidemiology):ti,ab,kw OR (Epidemiologies):ti,ab,kw | 230635 |
| #13 | #11 or #12 | 230635 |
| #14 | #7 and #10 and #13 | 141 |

3.Embase

| Search number | Query | Results |
| --- | --- | --- |
| #1 | 'minor (person)'/exp | 951 |
| #2 | 'minor'/exp OR minor OR child:ab, ti OR children:ab, ti OR juvenile:ab, ti OR childhood:ab, ti OR adolescent:ab, ti OR adolescents:ab, ti OR adolescence:ab,ti OR teens:ab,ti OR teen:ab,ti OR teenagers:ab,ti OR teenager:ab,ti OR youth:ab,ti OR youths:ab,ti OR younger:ab,ti OR pediatrics:ab,ti OR pediatric:ab,ti OR infants:ab,ti OR infant:ab,ti OR infantile:ab,ti | 3749580 |
| #3 | #1 or #2 | 3749580 |
| #4 | 'scoliosis'/exp | 44024 |
| #5 | scolioses OR kyphoscoliosis:ab,ti OR kyfoscoliosis:ab,ti | 3376 |
| #6 | #4 OR #5 | 44618 |
| #7 | 'prevalence'/exp | 1008147 |
| #8 | 'incidence'/exp OR incidence OR incidences:ab,ti OR epidemiology:ab,ti OR epidemiologies:ab,ti | 1770145 |
| #9 | #7 OR #8 | 2613554 |
| #10 | #3 AND #6 AND #9 | 2360 |

4.Web of science

| Search number | Query | Results |
| --- | --- | --- |
| #1 | Minors (TS) or Minor (TS) or child (TS) or children (TS) or juvenile (TS) or Childhood (TS) or adolescent (TS) or Adolescents (TS) or Adolescence (TS) or Teens (TS) or Teen (TS) or Teenagers (TS) or Teenager (TS) or Youth (TS) or Youths (TS) or Younger (TS) or Pediatrics (TS) or Pediatric (TS) or Infants (TS) or Infant (TS) or Infantile (TS) | 4697581 |
| #2 | (((TS=(Scoliosis)) OR TS=(Scolioses)) OR TS=(kyphoscoliosis)) OR TS=(kyfoscoliosis) | 28410 |
| #3 | ((((TS=(Prevalence)) OR TS=(Incidence)) OR TS=(Incidences)) OR TS=(Epidemiology)) OR TS=(Epidemiologies) | 2411701 |
| #4 | #1 AND #2 AND #3 | 2018 |
