# Supplementary material for: Prevalence of scoliosis in children and adolescents: a systematic review and meta-analysis
Source: Front Pediatr. 2024 Jul 23;12:1399049. doi: 10.3389/fped.2024.1399049 (PMC11300313; doi:10.3389/fped.2024.1399049)
Supplement: Supplementary Table S2 — Joanna Briggs Institute scale. [file Table2.docx]

| No | Author | Year | v1 | v2 | v3 | v4 | v5 | v6 | v7 | v8 | v9 |  |  |  | Yes | No | Unclear | Not applicable |
| --- | --- | --- | --- | --- | --- | --- | --- | --- | --- | --- | --- | --- | --- | --- | --- | --- | --- | --- |
| 1 | Yan Zou | 2022 | Yes | Yes | Yes | Yes | Yes | Yes | Yes | Yes | Yes |  |  | 1. Was the sample frame appropriate to address the target population?样本框架是否适用于目标人群？ | □ | □ | □ | □ |
| 2 | Sahyun Sung | 2021 | Yes | Yes | Yes | Yes | Yes | Yes | Yes | Yes | Yes |  |  | 2. Were study participants sampled in an appropriate way?研究参与者是否以适当的方式抽样？ | □ | □ | □ | □ |
| 3 | Lijin Zhou | 2022 | Yes | Yes | Yes | Yes | Yes | Yes | Yes | Yes | Yes |  |  | 3. Was the sample size adequate?样本量是否足够？ | □ | □ | □ | □ |
| 4 | Yu Zheng | 2016 | Yes | Yes | Yes | Yes | Yes | Yes | Yes | Yes | Yes |  |  | 4. Were the study subjects and the setting described in detail?是否详细描述了研究对象和环境？ | □ | □ | □ | □ |
| 5 | Flordeliza Yong, | 2009 | Yes | Yes | Yes | Yes | Yes | Yes | Yes | Yes | Yes |  |  | 5. Was the data analysis conducted with sufficient coverage of the identified sample? 进行的数据分析是否充分覆盖了确定的样本？ | □ | □ | □ | □ |
| 6 | Hurriyet Yılmaz | 2020 | Yes | Yes | Yes | Yes | Yes | Yes | Yes | Yes | Yes |  |  | 6. Were valid methods used for the identification of the condition? 是否使用了有效的方法来识别病情？ | □ | □ | □ | □ |
| 7 | Hee-Kit Wong | 2005 | Yes | Yes | Yes | Yes | Yes | Yes | Yes | Yes | Yes |  |  | 7. Was the condition measured in a standard, reliable way for all participants? 是否以标准、可靠的方式测量了所有参与者的病情？ | □ | □ | □ | □ |
| 8 | STIG WILLNER | 1982 | Yes | Yes | Yes | Yes | Yes | Yes | Yes | Yes | Yes |  |  | 8. Was there appropriate statistical analysis? 是否进行了适当的统计分析？ | □ | □ | □ | □ |
| 9 | Fei Wang | 2021 | Yes | Yes | Yes | Yes | Yes | Yes | Yes | Yes | Yes |  |  | 9. Was the response rate adequate, and if not, was the low response rate managed appropriately?响应率是否足够，如果不足够，低响应率是否得到适当管理？ | □ | □ | □ | □ |
| 10 | Masaki Ueno | 2011 | Yes | Yes | Yes | Yes | Yes | Yes | Yes | Yes | Yes |  |  |  |  |  |  |  |
| 11 | J. STIRLING | 1996 | Yes | Yes | Yes | Yes | Yes | Yes | Yes | Yes | Yes |  |  |  |  |  |  |  |
| 12 | Hemender Singh | 2022 | Yes | Yes | Yes | Yes | Yes | Yes | Yes | Yes | Yes |  |  |  |  |  |  |  |
| 13 | Comron Saifi | 2012 | Yes | Yes | No | Yes | Yes | Yes | Yes | Yes | Yes |  |  |  |  |  |  |  |
| 14 | Patrı´cia Jundi Penha | 2018 | Yes | Yes | Yes | Yes | Yes | Yes | Yes | Yes | Yes |  |  |  |  |  |  |  |
| 15 | Zdenko Ostoji} | 2005 | Yes | Yes | Yes | Yes | Yes | Yes | Yes | Yes | Yes |  |  |  |  |  |  |  |
| 16 | Lenice Sberse Nery | 2010 | Yes | Yes | Yes | Yes | Yes | Yes | Yes | Yes | Yes |  |  |  |  |  |  |  |
| 17 | Sepehr Moalej | 2018 | Yes | Yes | No | Yes | Yes | Yes | Yes | Yes | Yes |  |  |  |  |  |  |  |
| 18 | Beatriz Minghelli | 2014 | Yes | Yes | Yes | Yes | Yes | Yes | Yes | Yes | Yes |  |  |  |  |  |  |  |
| 19 | Jin-Young Lee | 2014 | Yes | Yes | Yes | Yes | Yes | Yes | Yes | Yes | Yes |  |  |  |  |  |  |  |
| 20 | Sombat Kunakornsawat MD | 2006 | Yes | Yes | Yes | Yes | Yes | Yes | Yes | Yes | Yes |  |  |  |  |  |  |  |
| 21 | Zahed Safikhani | 2006 | Yes | Yes | Yes | Yes | Yes | Yes | Yes | Yes | Yes |  |  |  |  |  |  |  |
| 22 | Komang-Agung IS | 2017 | Yes | Yes | Yes | Yes | Yes | Yes | Yes | Yes | Yes |  |  |  |  |  |  |  |
| 23 | Janani | 2019 | Yes | Yes | Yes | Yes | Yes | Yes | Yes | Yes | Yes |  |  |  |  |  |  |  |
| 24 | Fuli Huang | 2019 | Yes | Yes | Yes | Yes | Yes | Yes | Yes | Yes | Yes |  |  |  |  |  |  |  |
| 25 | Miao Hu | 2022 | Yes | Yes | Yes | Yes | Yes | Yes | Yes | Yes | Yes |  |  |  |  |  |  |  |
| 26 | Fan Hengwei | 2016 | Yes | Yes | Yes | Yes | Yes | Yes | Yes | Yes | Yes |  |  |  |  |  |  |  |
| 27 | Mohammadreza Etemadifar | 2020 | Yes | Yes | Yes | Yes | Yes | Yes | Yes | Yes | Yes |  |  |  |  |  |  |  |
| 28 | Murat Şakir Ekşi | 2019 | Yes | Yes | Yes | Yes | Yes | Yes | Yes | Yes | Yes |  |  |  |  |  |  |  |
| 29 | Qing Du | 2014 | Yes | Yes | Yes | Yes | Yes | Yes | Yes | Yes | Yes |  |  |  |  |  |  |  |
| 30 | J. S. DARUWALLA, | 1985 | Yes | Yes | Yes | Yes | Yes | Yes | Yes | Yes | Yes |  |  |  |  |  |  |  |
| 31 | Milla Gabriela Belarmino Dantas | 2021 | Yes | Yes | Yes | Yes | Yes | Yes | Yes | Yes | Yes |  |  |  |  |  |  |  |
| 32 | Kevin Bondar, B.S | 2021 | Yes | Yes | Yes | Yes | Yes | Yes | Yes | Yes | Yes |  |  |  |  |  |  |  |
