# Supplementary material for: Prevalence of scoliosis in children and adolescents: a systematic review and meta-analysis
Source: Front Pediatr. 2024 Jul 23;12:1399049. doi: 10.3389/fped.2024.1399049 (PMC11300313; doi:10.3389/fped.2024.1399049)
Supplement: Supplementary Table S3 — Independent risk factors. [file Table3.docx]

| No | Author | Year | Factors | Value | OR | low | up | lnor | selnor |
| --- | --- | --- | --- | --- | --- | --- | --- | --- | --- |
| 1 | Yan Zou | 2022 | Age | NA | 1.145 | 1.128 | 1.162 | 0.135404637 | 0.007575639 |
| 1 | Yan Zou | 2022 | Gender | Male | 1.118 | 1.016 | 1.23 | 0.111541375 | 0.048760413 |
| 1 | Yan Zou | 2022 | Living area | Urban area | 1.04 | 0.942 | 1.148 | 0.039220713 | 0.050451863 |
| 1 | Yan Zou | 2022 | Low weight | Yes | 1.48 | 1.25 | 1.751 | 0.392042088 | 0.085980485 |
| 2 | Lijin Zhou | 2022 | Age | NA | 0.95 | 0.917 | 0.983 | -0.051293294 | 0.017730012 |
| 2 | Lijin Zhou | 2022 | Gender | Male | 0.378931413 | 0.301386377 | 0.476417342 | -0.970400058 | 0.116811518 |
| 2 | Lijin Zhou | 2022 | BMI | <18.5 | 2.454 | 1.94 | 3.104 | 0.897719346 | 0.119898885 |
| 2 | Lijin Zhou | 2022 | Altitudes of residence | ≥4,500 m | 1.644 | 1.21 | 2.235 | 0.497132297 | 0.156535936 |
| 2 | Lijin Zhou | 2022 | Living area | Urban area | 0.509683996 | 0.4 | 0.649350649 | -0.673964361 | 0.12359906 |
| 2 | Lijin Zhou | 2022 | Appetite | Good | 1 | 1 | 1 | 0 | 0 |
| 2 | Lijin Zhou | 2022 | Appetite | General | 1.835 | 1.422 | 2.368 | 0.607044482 | 0.130097292 |
| 2 | Lijin Zhou | 2022 | Appetite | Bad | 3.017 | 2.258 | 4.031 | 1.10426296 | 0.147840566 |
| 2 | Lijin Zhou | 2022 | Myopia | No | 1 | 1 | 1 | 0 | 0 |
| 2 | Lijin Zhou | 2022 | Myopia | Unclear | 0.937 | 0.746 | 1.177 | -0.065071997 | 0.11632615 |
| 2 | Lijin Zhou | 2022 | Myopia | Yes | 0.574 | 0.418 | 0.789 | -0.555125883 | 0.162062472 |
| 2 | Lijin Zhou | 2022 | Sleep time | <8 h | 2.06 | 1.595 | 2.66 | 0.722705983 | 0.130472548 |
| 2 | Lijin Zhou | 2022 | Daily exercise time | <1 h | 1.407 | 0.989 | 2.002 | 0.341459778 | 0.179899905 |
| 2 | Lijin Zhou | 2022 | Cognition of scoliosis | Known | 1 | 1 | 1 | 0 | 0 |
| 2 | Lijin Zhou | 2022 | Cognition of scoliosis | Have heard | 2.659 | 1.376 | 5.135 | 0.977950112 | 0.335943649 |
| 2 | Lijin Zhou | 2022 | Cognition of scoliosis | Never known | 3.192 | 1.69 | 6.029 | 1.16064768 | 0.324452202 |
| 3 | Flordeliza Yong | 2009 | Age (y) | 9 | 1 | 1 | 1 | 0 | 0 |
| 3 | Flordeliza Yong | 2009 | Age (y) | 11-12 | 1.7 | 1.1 | 2.4 | 0.530628251 | 0.19902004 |
| 3 | Flordeliza Yong | 2009 | Age (y) | 12-13 | 2.2 | 1.2 | 3.3 | 0.78845736 | 0.258061457 |
| 3 | Flordeliza Yong | 2009 | Menarche | No menarche yet (9-12 y) | 1 | 1 | 1 | 0 | 0 |
| 3 | Flordeliza Yong | 2009 | Menarche | Early menarche (<13 y) | 1.5 | 1.1 | 1.9 | 0.405465108 | 0.139424415 |
| 3 | Flordeliza Yong | 2009 | Menarche | Late menarche (>13 y) | 1.5 | 0.9 | 2.4 | 0.405465108 | 0.250211544 |
| 3 | Flordeliza Yong | 2009 | Standing height (cm) | >150 | 1 | 0.8 | 1.3 | 0 | 0.123854035 |
| 3 | Flordeliza Yong | 2009 | Sitting height (cm) | >75 | 1.1 | 0.8 | 1.5 | 0.09531018 | 0.160359352 |
| 3 | Flordeliza Yong | 2009 | Ratio of upper to lower segments | <1.2 | 1.6 | 1.1 | 2.3 | 0.470003629 | 0.188162996 |
| 3 | Flordeliza Yong | 2009 | BMI | Healthy and overweight (>18.5) | 1.5 | 1.2 | 1.8 | 0.405465108 | 0.103434977 |
| 3 | Flordeliza Yong | 2009 | Race | Chinese | 1 | 1 | 1 | 0 | 0 |
| 3 | Flordeliza Yong | 2009 | Race | Malay | 0.7 | 0.5 | 1 | -0.356674944 | 0.17682326 |
| 3 | Flordeliza Yong | 2009 | Race | Indian | 1.1 | 0.7 | 1.7 | 0.09531018 | 0.226352856 |
| 3 | Flordeliza Yong | 2009 | Race | Others | 0.5 | 0.2 | 1.2 | -0.693147181 | 0.457081497 |
| 4 | Miao Hu | 2022 | Gender | Male | 0.612745098 | 0.397772474 | 0.944287063 | -0.489806257 | 0.22054848 |
| 4 | Miao Hu | 2022 | Sitting time | NA | 1.193 | 1.09 | 1.306 | 0.176471143 | 0.046120238 |
| 4 | Miao Hu | 2022 | Dancing time | NA | 1.559 | 1.178 | 2.072 | 0.44404459 | 0.144055163 |
| 5 | Kevin Bondar | 2021 | Sex | Male | 0.432900433 | 0.392156863 | 0.476190476 | -0.837247525 | 0.049529596 |
| 5 | Kevin Bondar | 2021 | Race/ethnicity | Asian/Pacific Islander | 1.54 | 1.33 | 1.79 | 0.431782416 | 0.075774663 |
| 5 | Kevin Bondar | 2021 | Race/ethnicity | Black | 0.9 | 0.75 | 1.08 | -0.105360516 | 0.093021202 |
| 5 | Kevin Bondar | 2021 | Race/ethnicity | White | 1.32 | 1.19 | 1.47 | 0.277631737 | 0.053905381 |
| 5 | Kevin Bondar | 2021 | Race/ethnicity | Other/Unknown | 0.77 | 0.63 | 0.93 | -0.261364764 | 0.099353257 |
| 5 | Kevin Bondar | 2021 | Race/ethnicity | Hispanic | 1 | 1 | 1 | 0 | 0 |
| 5 | Kevin Bondar | 2021 | Age group | Age 10-17 | 20.73 | 16.83 | 25.54 | 3.031581926 | 0.106398684 |
| 5 | Kevin Bondar | 2021 | BMI | Underweight | 1.5 | 1.16 | 1.94 | 0.405465108 | 0.131190808 |
| 5 | Kevin Bondar | 2021 | BMI | Normal weight | 1 | 1 | 1 | 0 | 0 |
| 5 | Kevin Bondar | 2021 | BMI | Overweight | 0.51 | 0.45 | 0.58 | -0.673344553 | 0.064739929 |
| 5 | Kevin Bondar | 2021 | BMI | Obesity | 0.35 | 0.29 | 0.42 | -1.049822124 | 0.094483109 |
| 5 | Kevin Bondar | 2021 | BMI | Extremely Obesity | 0.3 | 0.23 | 0.4 | -1.203972804 | 0.141169704 |
| 6 | Yu Zheng | 2016 | Sex | Male | 0.1 | 0.02 | 0.02 | -2.302585093 | 0 |
| 6 | Yu Zheng | 2016 | Age | NA | NA | NA | NA | NA | NA |
| 6 | Yu Zheng | 2016 | Distance from home to school | NA | 0.56 | 0.17 | 2.51 | -0.579818495 | 0.686795815 |
| 6 | Yu Zheng | 2016 | Schoolbag type | Single-shoulder bag | 9.04 | 1.53 | 53.44 | 2.201659174 | 0.906451988 |
| 6 | Yu Zheng | 2016 | Schoolbag type | Backpack | 0.23 | 0.04 | 1.19 | -1.46967597 | 0.865517636 |
| 6 | Yu Zheng | 2016 | Schoolbag type | Trolley case | 0.94 | 0.09 | 9.57 | -0.061875404 | 1.190453779 |
| 6 | Yu Zheng | 2016 | Weight of schoolbag | NA | 1.1 | 0.95 | 1.26 | 0.09531018 | 0.072042096 |
| 6 | Yu Zheng | 2016 | Activity times | Extracurricular sports | 0.99 | 0.8 | 1.21 | -0.010050336 | 0.105552018 |
| 6 | Yu Zheng | 2016 | Activity times | Extracurricular activities | 1.48 | 1.01 | 2.17 | 0.392042088 | 0.195096132 |
| 6 | Yu Zheng | 2016 | Activity times | Study at home | 0.93 | 0.79 | 1.1 | -0.072570693 | 0.08444707 |
| 6 | Yu Zheng | 2016 | Activity times | Computer use at home | 1.17 | 1.01 | 1.36 | 0.157003749 | 0.075901625 |
| 6 | Yu Zheng | 2016 | Sleeping posture | Prone | 0.26 | 0.03 | 2.5 | -1.347073648 | 1.128277712 |
| 6 | Yu Zheng | 2016 | Sleeping posture | Left lateral | 0.72 | 0.13 | 4.03 | -0.328504067 | 0.876017144 |
| 6 | Yu Zheng | 2016 | Sleeping posture | Right lateral | 2.99 | 0.53 | 16.87 | 1.095273387 | 0.882758972 |
| 6 | Yu Zheng | 2016 | Calcium and milk supplements | Calcium supplement (baby) | 1.49 | 0.19 | 11.56 | 0.39877612 | 1.04803114 |
| 6 | Yu Zheng | 2016 | Calcium and milk supplements | Calcium supplement (now) | 0.65 | 0.06 | 6.94 | -0.430782916 | 1.211916452 |
| 6 | Yu Zheng | 2016 | Calcium and milk supplements | Milk supplement | 0.62 | 0.1 | 3.71 | -0.478035801 | 0.921841064 |
| 6 | Yu Zheng | 2016 | Annual income/1,000 | NA | 0.98 | 0.96 | 0.99 | -0.020202707 | 0.007849913 |
| 6 | Yu Zheng | 2016 | Eutocia | NA | 0.34 | 0.05 | 2.16 | -1.078809661 | 0.960673596 |
| 6 | Yu Zheng | 2016 | BMI | NA | 0.03 | 0 | 2.54 | -3.506557897 | #NUM! |
| 7 | Patrı´cia Jundi Penha | 2018 | Sex | Male | 0.212766 | 0.081967 | 0.555556 | -1.547562309 | 0.488176706 |
| 7 | Patrı´cia Jundi Penha | 2018 | 13–14 Years of age | NA | 2.2 | 1 | 4.8 | 0.78845736 | 0.400157122 |
| 7 | Patrı´cia Jundi Penha | 2018 | Height >1.56 m | NA | 1.7 | 0.8 | 3.7 | 0.530628251 | 0.390682748 |
